# Hidden in Plain Sight: Unmasking Hairy Cell Leukemia Through Structured Clinical Reasoning

**DOI:** 10.7759/cureus.100803

**Published:** 2026-01-05

**Authors:** Luís D Veiga, Inês F Teixeira, Joana B Vaz, Paula Leite, Joana Gonçalves

**Affiliations:** 1 Internal Medicine, ULS Póvoa de Varzim/Vila do Conde, Póvoa de Varzim, PRT; 2 Clinical Pathology, ULS Póvoa de Varzim/Vila do Conde, Póvoa de Varzim, PRT

**Keywords:** clinical pathology, cytopenia, diagnostic reasoning, differential diagnosis, hairy cell leukemia, indolent b-cell neoplasm, multidisciplinary care, peripheral smear, thrombocytopenia

## Abstract

Hairy cell leukemia (HCL) is a rare, indolent B-cell neoplasm that often presents with nonspecific laboratory findings, which can delay diagnosis. We report the case of a 75-year-old male with incidentally detected isolated thrombocytopenia and relative lymphocytosis. Peripheral blood smear showed lymphoid cells with cytoplasmic projections suggestive of hairy cells. Flow cytometry confirmed a dominant clonal population consistent with classical HCL, along with a small CD5+/CD23+ monoclonal B-cell population compatible with a chronic lymphocytic leukemia-like clone. Abdominal CT also revealed a renal lesion suspicious for carcinoma. This incidental finding led to the prioritization of urological intervention, illustrating how multidisciplinary management is coordinated when concurrent malignancies are present. The patient remains asymptomatic and under active surveillance for both conditions. Subtle hematologic abnormalities may indicate early stages of HCL, and internists play a crucial role in recognizing these early signs and initiating targeted diagnostic evaluation.

## Introduction

Hairy cell leukemia (HCL) is a rare, mature B-cell lymphoproliferative disorder accounting for approximately 2% of all leukemias [1]. It is characterized by small-to-medium-sized B lymphocytes with fine cytoplasmic projections (“hairy cells”) and a distinctive immunophenotype typically showing bright CD20 expression along with CD11c, CD103, and CD123 [2]. The near-universal presence of the BRAF V600E mutation in classical HCL has further refined diagnostic classification and guided targeted therapeutic strategies [2]. Despite these well-defined features, HCL often presents insidiously. Cytopenias - sometimes isolated - and subtle or incidental abnormalities on routine blood tests may be the earliest signs, leading to frequent misinterpretation as benign or age-related changes [3,4].

Maintaining a high index of diagnostic suspicion in the setting of unexplained hematologic abnormalities is therefore essential. Peripheral blood smear evaluation and immunophenotyping remain crucial for early recognition, while timely referral to hematology facilitates appropriate surveillance and clinical decision-making [2,5]. This report underscores the pivotal role of internal medicine in detecting rare hematologic malignancies such as HCL, which may be masked by seemingly minor laboratory abnormalities like isolated thrombocytopenia and relative lymphocytosis, thereby enabling prompt diagnostic clarification and necessary multidisciplinary coordination.

## Case presentation

A 75-year-old retired male, living independently with his wife, was referred to Internal Medicine for evaluation of persistent abnormalities on routine blood tests identified during primary care follow-up. He reported no symptoms, including fatigue, fever, weight loss, night sweats, infections, bleeding, or abdominal discomfort. His medical history included dyslipidemia managed with dietary measures alone, multiple simple bilateral renal cysts under surveillance, and benign prostatic hyperplasia treated surgically five years previously with good outcomes. He was a former smoker with more than 50 years of abstinence, consumed alcohol occasionally, took no regular medications other than a nutritional supplement, and had no known drug allergies. Vital signs were normal. Physical examination was unremarkable, with no palpable lymphadenopathy, hepatomegaly, or splenomegaly.

Initial laboratory evaluation revealed preserved hemoglobin levels, normal total leukocyte count with relative lymphocytosis and relative neutropenia, and thrombocytopenia. Inflammatory, renal, hepatic, thyroid, nutritional, autoimmune, and viral studies were within normal limits or negative, which ruled out common secondary causes of cytopenias (Table 1).

**Table 1:** Baseline hematologic and biochemical parameters AST: aspartate aminotransferase; ALT: alanine aminotransferase; HBV: hepatitis B virus; HCV: hepatitis C virus

Parameter	Result	Reference range	Interpretation
Hemoglobin	14.9 g/dL	13.0–17.0 g/dL	Normal
White blood cells	5.09 ×10⁹/L	4.0–11.0 ×10⁹/L	Normal count with differential changes
Neutrophils	27%	40–70%	Relative neutropenia
Lymphocytes	58%	20–45%	Relative lymphocytosis
Platelets	76 ×10³/µL	150–400 ×10³/µL	Thrombocytopenia
C-reactive protein	0.10 mg/dL	<0.5 mg/dL	No inflammation
ESR	6 mm/h	<20 mm/h	Normal
Creatinine	1.01 mg/dL	0.7–1.2 mg/dL	Normal renal function
AST/ALT	Normal	<40 U/L	No hepatic injury
Bilirubin	Normal	<1.2 mg/dL	No hemolysis
Vitamin B12	333 pg/mL	200–900 pg/mL	Normal
Folate	>20 ng/mL	>4 ng/mL	Normal
Ferritin	509 ng/mL	24–336 ng/mL	Mild elevation
Serum iron	97 µg/dL	60–170 µg/dL	Normal
Autoimmune screen	Negative	—	Excludes immune causes
HIV serology	Negative	—	Excludes viral cytopenias
HBV serology	Non-immune	—	Vaccination started
HCV serology	Negative	—	Excludes viral cytopenias

**Figure 1:**
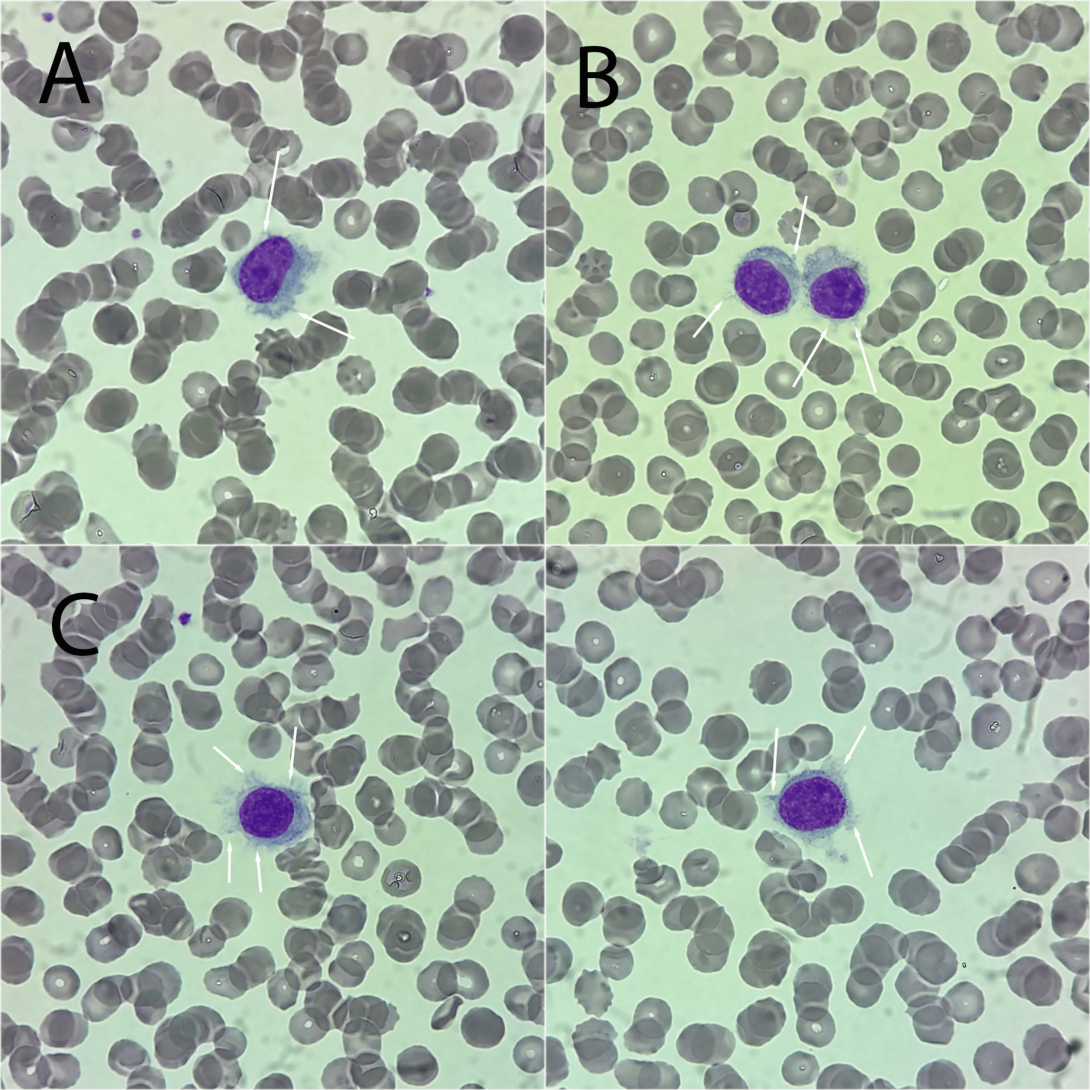
Peripheral blood smear demonstrating classical hairy cells Wright–Giemsa stain showing circulating hairy cells with circumferential cytoplasmic projections (arrows), ×1000. Panels A-D show representative cells from different fields

Peripheral blood smear demonstrated atypical lymphoid cells with pale cytoplasm and fine cytoplasmic projections consistent with hairy cells (Figure 1).

Flow cytometry of peripheral blood further revealed a dominant monoclonal B-cell population immunophenotypically consistent with classical HCL and a smaller CD5+/CD23+ kappa-restricted clone compatible with a chronic lymphocytic leukemia (CLL)-like population (Table 2).

**Table 2:** Flow cytometry analysis supporting HCL diagnosis HCL: hairy cell leukemia; CLL: chronic lymphocytic leukemia

Parameter	Result	Reference range	Interpretation
Immunophenotypic evaluation			
Dominant B-cell clone	74%	—	Classical HCL phenotype
Small B-cell clone	12%	—	CLL-like phenotype
Polyclonal B-cells	14%	—	Residual normal

**Figure 2:**
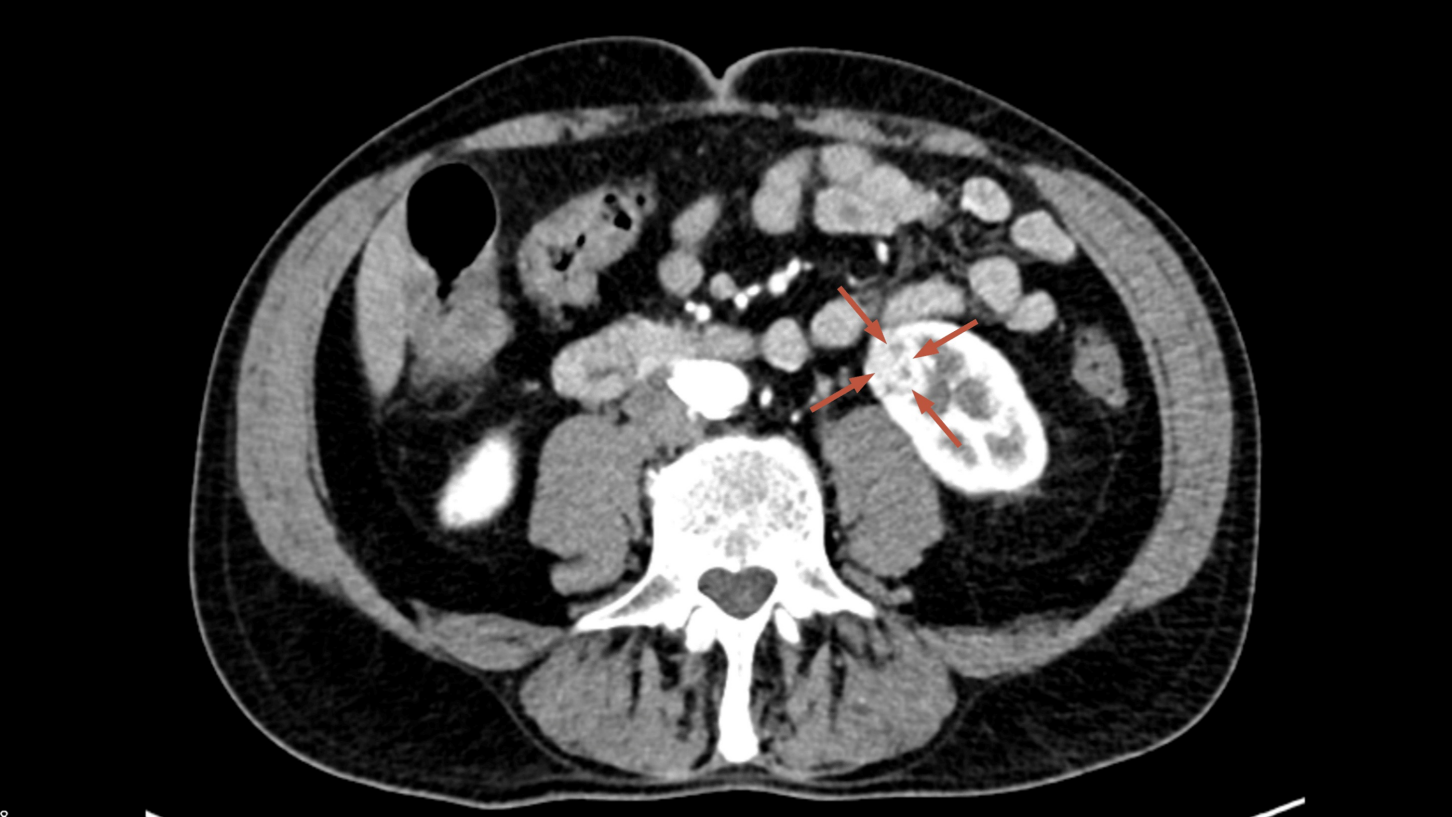
Contrast-enhanced CT of the abdomen showing a left renal mass Contrast-enhanced axial CT showing an 18 × 22 mm enhancing lesion in the mid-portion of the left kidney (arrows), containing areas of macroscopic fat and radiologically consistent with a renal cell carcinoma. There is no invasion of the renal sinus or surrounding structures. Additional simple cortical and parapelvic renal cysts are noted bilaterally without suspicious features CT: computed tomography

CT of the abdomen and pelvis incidentally identified an 18 × 22 mm enhancing renal lesion with macroscopic fat, highly suspicious for renal cell carcinoma, without sinus or adjacent structure invasion (Figure 2). A tiny hepatic cyst and multiple benign renal cysts were also noted.

Based on the hematologic findings and immunophenotypic profile, a diagnosis of classical HCL with a coexisting small CLL-like B-cell clone was established. Given the absence of anemia, splenomegaly, infectious complications, or clinically significant symptoms, an active surveillance approach was recommended. A bone marrow biopsy was initially deferred, as the diagnosis was supported by peripheral blood morphology and flow cytometry and would not alter immediate management. The patient was referred to Hematology for follow-up and to Urology for evaluation and potential surgical management of the renal lesion, with curative intervention prioritized before any requirement for systemic therapy. A bone marrow biopsy was subsequently performed at a specialized hemato-oncology center. However, the results were unavailable at the time of preparation of this report and did not affect the initial clinical approach. At the time of writing, the patient remains asymptomatic and clinically stable, with scheduled follow-up visits in both specialties.

## Discussion

The timely diagnosis of rare malignancies is often complicated by nonspecific presentations and subtle initial clues, a diagnostic hurdle well-documented across medical specialties. HCL typically follows an indolent clinical course, but its early manifestations may be subtle and contribute to delayed recognition [2,4]. As illustrated in this case, isolated thrombocytopenia with relative lymphocytosis - a pattern distinct from the classic triad of pancytopenia and splenomegaly - may represent the only initial hematologic clue. Such findings warrant thorough evaluation, beginning with peripheral smear review, as morphological identification of the characteristic cytoplasmic projections remains a critical diagnostic trigger [2].

Flow cytometry confirms the diagnosis, revealing the canonical immunophenotype of classical HCL [2]. The detection of a concurrent CLL-like monoclonal B-cell population exemplifies composite lymphoproliferative disorders, a phenomenon increasingly recognized with the use of sensitive immunophenotyping techniques [6]. While the prognostic significance of such coexisting clonal populations in this context remains uncertain, their identification underscores the importance of comprehensive flow cytometric analysis to prevent diagnostic misclassification and to inform appropriate monitoring strategies.

The management of asymptomatic classical HCL is guided by the degree of cytopenias, splenic involvement, and infectious risk [4]. Given the patient’s preserved hemoglobin levels, absence of splenomegaly, and stable clinical condition, an active surveillance strategy was considered the most appropriate approach. When treatment is indicated, purine analogs such as cladribine remain the standard first-line therapy, achieving high complete remission rates and prolonged survival [1]. The incidental renal mass identified in this case required prioritization of urologic evaluation and potentially curative intervention before considering immunosuppressive therapy. This scenario exemplifies the importance of multidisciplinary coordination orchestrated by internal medicine, which is essential to triage interventions and ensure timely and balanced decision-making when concurrent malignancies are identified.

Finally, the management of patients with indolent hematologic malignancies extends beyond initial diagnosis and treatment planning. It mandates structured, long-term surveillance to monitor for disease progression and manage emergent complications, a principle underscored by reports of severe and unexpected events such as spontaneous splenic or renal rupture in patients with other chronic leukemias [7]. In the present case, vigilant supervision remains essential not only for monitoring the course of HCL but also for overseeing follow-up of the concurrent, potentially curative renal malignancy.

## Conclusions

Classical HCL may remain hidden in plain sight, presenting solely with minor hematologic abnormalities and without splenomegaly or constitutional symptoms. This report illustrates how careful interpretation of routine laboratory findings, together with early peripheral smear review and directed flow cytometry, enables the timely unmasking of this rare malignancy before clinical deterioration occurs. It also underscores the pivotal role of internal medicine in structured diagnostic reasoning and in coordinating multidisciplinary care when concurrent conditions are identified.
